# Health care providers’ attitudes towards transfer and transition in young persons with long term illness- a web-based survey

**DOI:** 10.1186/s12913-017-2192-5

**Published:** 2017-04-11

**Authors:** Carina Sparud-Lundin, Malin Berghammer, Philip Moons, Ewa-Lena Bratt

**Affiliations:** 1 Institute of Health and Care Sciences, The Sahlgrenska Academy, University of Gothenburg, Box 457, SE- 405 30 Gothenburg, Sweden; 2 Department of Health Science, University West, SE-461 32 Trollhättan, Sweden; 3 Centre for Person-Centred Care (GPCC), University of Gothenburg, Box 457, SE- 405 30 Gothenburg, Sweden; 4 KU Leuven Department of Public Health and Primary Care, Kapucijnenvoer 35, Box 7001, BE-3000 Leuven, Belgium; 5 Department of Pediatric Cardiology, The Queen Silvia Children’s Hospital, SE-416 85 Gothenburg, Sweden

**Keywords:** Transition, Young persons, Chronic illness, Health care professionals, Attitudes

## Abstract

**Background:**

Transition programs in health care for young persons with special health care needs aim to maximize lifelong functioning. Exploring health care professionals’ perspective may increase the possibility of successful implementation of transition programs. The aim was to survey health care professionals’ attitudes towards components and barriers on transition and transfer in young people with long-term medical conditions with special health care needs.

**Methods:**

A cross-sectional web-based survey was sent by e-mail to 529 physicians and nurses in Swedish pediatric and adult outpatient clinics. Response rate was 38% (*n* = 201). The survey consisted of 59 questions regarding different aspects of components and barriers on transition and transfer. Descriptive statistics were computed to summarize demographic data and categorized responses. The Chi square test was used for comparison between proportions of categories.

**Results:**

Most respondents agreed on the destinations of care for adolescents within their specialty. Age and psychosocial aspects such as maturity and family situations were considered the most important initiators for transfer. Joint meeting with the patient (82%); presence of a transition coordinator (76%) and a written individualized transfer plan (55%) were reported as important transition components. Pediatric care professionals found the absence of a transition coordinator to be more of a transition barrier than adult care professionals (*p* = 0.018) and also a more important transfer component (*p* = 0.017). Other barriers were lack of funding (45%) and limited clinical space (19%). Transition programs were more common in university hospitals than in regional hospitals (12% vs 2%, *p* = <0.001) as well as having a transition coordinator (12% vs 3%, *p* = 0.004).

**Conclusion:**

The findings highlight a willingness to work on new transition strategies and provide direction for improvement, taking local transition components as well as potential barriers into consideration when implementing future transition programs. Some differences in attitudes towards transitional care remain among pediatric and adult care professionals.

## Background

Medical improvements in recent decades have resulted in an increasing group of young persons with long-term medical conditions who are in need of special health care throughout their lives [1]. In 2002, a consensus statement by the American Academy of Pediatrics, the American Academy of Family Physicians, and the American College of Physicians-American Society of Internal Medicine was published, stating the importance of supporting and facilitating the transition of adolescents with special health care needs into adulthood. This statement represents the shared perspectives of health care professionals, families, young people, researchers, and policy-makers [1]. Successful transition involves the engagement and participation of the entire medical team (physicians, nurses, care coordinators), the family and other caregivers, and the young person collaborating in a positive and mutually respectful relationship [2].

Transition programs for young persons with special health care needs aim to maximize lifelong functioning and potential. When developing a transition program, according to the comprehensive framework for health promotion program development (intervention mapping) [3], it is crucial to perform a needs assessment to scrutinize all perspectives and contexts involved. Such perspectives include that of the young persons and their social network, hospital and health administrators, and health policy makers. Moreover, health care professionals’ perspectives are also essential in order to understand the context in which a future transition program will be executed [3], since clinicians will be the executors of such a transition program. However, until now, knowledge about health professionals’ attitudes, experiences and organizational factors of transitional care within the field of young persons with chronic disease are limited [2, 4–6]. It is essential to take health care professionals’ perspectives and context into consideration when developing a transition program because it will increase the possibility of successful implementation. The aim of the present study therefore was to survey health care professionals’ attitudes towards components and barriers of transition and transfer in young people with long-term medical conditions and special health-care needs.

## Methods

A national survey in Sweden was undertaken in order to capture a broad cross-section of health care professionals (HCP) involved in transition care. The STROBE guidelines were adhered to.

### Setting, procedure and participants

In Sweden, where the current study took place, pediatric patients with long term medical conditions are generally transferred to adult care at the age of 18. Inclusion criteria were HCP in pediatric and adult outpatient clinics with specialized care in the area of endocrinology/diabetes, oncology, congenital heart disease (CHD) and inflammatory bowel disease (IBD). All university hospitals in Sweden (*n* = 7) and collaborating hospitals (*n* = 19) were invited to participate in the present survey. To gain authorization to contact the health care professionals, we first sent an invitation to participate to the head of department for pediatric or adult care settings within the area of endocrinology/diabetes, oncology, CHD and IBD. When authorization was confirmed by the head of departments in a response mail to our invitation, we were provided e-mail addresses of all relevant physicians and nurses working at the clinic. In this way, we got access to the email addresses of 529 HCP. The accessible HCPs received the invitation to participate including a link for access to the questionnaire ATTITUDE distributed via Webropol^©^ in March 2016. After two weeks, an email reminder was sent to non-responders, followed by two additional reminders three weeks after each other. Data collection was closed in June 2016. Information about the study was included in the e-mail to the potential participants, stating that participation was voluntary and informed consent was considered to be obtained through the act of responding.

The HCPs (*n* = 529) contacted at these outpatient clinics were clinicians (physicians and nurses) involved in transition care. In total, 335 professionals from the pediatric setting (63%) and 194 professionals from the adult setting (37%) were invited to participate. The gender distribution of invited participants was 181 men (34%) and 348 women (66%). The total response rate was 201 (38%). Demographic and professional characteristics are presented in Table 1. The majority (50.5%) of the participants that responded were between 50–65 years and most were females (76%). Employees at university hospitals and in the pediatric setting were also in the majority. Specialties such as endocrinology/diabetes, oncology, CHD and IBD, were all represented, with most respondents from diabetes, endocrinology and CHD.

**Table 1:** Characteristics of participants (*n* = 201) and settings

Female gender	153 (76.1%)
Age	
30-29	20 (10%)
40-49	74 (37%)
50-65	101 (50.5%)
>65	5 (2.5%)
Education	
Medical doctor	107 (54%)
Nurse	86 (43.4%)
Other^a^	5 (2.5%)
Employment	
University hospital	116 (58%)
Regional hospital	80 (40%)
Both	2 (1%)
Other	2 (1%)
Setting	
Pediatric care	139 (70.2%)
Adult care	58 (29.3%)
Both	1 (0.5%)
Specialty^b^	
Diabetes	61 (34.1%)
Congenital heart disease	57 (31.8%)
Oncology	23 (12.9%)
Gastroenterology	24 (13.4%)
Endocrinology	46 (25.7%)

^a^the questionnaire was meant to be sent to nurses and physicians only ^b^several options possible

### Measurements

For the purpose of this study, we developed the ‘Attitudes to Transition and Transfer Instrument To be Used in aDolescent carE’ (ATTITUDE). This instrument is based on a questionnaire developed by Hilderson et al. [6], initially developed for the area of pediatric rheumatology. We modified the rheumatology-oriented questions in the former questionnaire to questions of a more generic nature. This was done in order to capture attitudes of professionals in various medical specialties, from pediatric as well as adult settings.

The questions in ATTITUDE were initially developed in English and tested for content validity according to the recommendations proposed by Polit & Beck [7]. Fifteen international adolescent health researchers and clinical experts in pediatric and adult settings (endocrinology/diabetes, oncology, CHD and IBD) assisted in validating the current questionnaire. We evaluated content validity by calculating a content validity index at the summary score level, S-CVI/Ave, the average of the proportion of items rated as ‘relevant’ by the experts. The S-CVI/Ave of the ATTITUDE was 0.88. The generally accepted cutoff is 0.90 or higher, and the content validity was considered to be almost excellent. An additional measurement for content validity that adjusts for chance was calculated: a kappa for multiple raters. Forty-nine items had a kappa score > 0.74 (excellent agreement), six items had a kappa score 0.60–0.74 (good agreement) and 4 items showed a kappa score < 0.60 (fair agreement). Of the four items indicating fair agreement, three were removed from the questionnaire.

The English version was translated to Swedish according to the recommendations from Wild et al. [8]. The translation process includes forward- backward translations, reviews, harmonization, testing face validity and cultural adaption by cognitive debriefing and finalizing to a final version.

The final Swedish version of ATTITUDE consists of 59 items over 8 domains; ‘destinations of care’ (4 items), ‘initiators for transfers’ (8 items), ‘transfer communications with adult health care’ (6 items), ‘participants in transition in your specialty’ (11 items), ‘transition barriers’ (8 items), ‘patient education’ (9 items), ‘transition components’ (10 items) and ‘transition involvement’ (3 items). Respondents were asked to rate their level of agreement on a Likert scale, ranging from strongly disagree to strongly agree. Three additional questions on transition issues were included in the Swedish version, as well as three open questions where respondents can describe with their own words. Quotes from the written comments are provided to illustrate participants’ opinions. Questions related to characteristics of participants in terms of age, gender, profession and work experience were included at the end of the questionnaire.

### Data analysis

Data were analyzed by means of statistical tools included in Webropol ^©^ and exported to SPSS (Statistics for Windows version 22. Armonk, NY: IBM Corp.) for comparative analyses. Descriptive statistics were computed to summarize demographic data and categorized responses. Data on nominal and ordinal levels were reported as percentages and absolute numbers. The response options were dichotomized into: disagree/strongly disagree and agree/strongly agree. When the assumptions were met, the Chi square test was used for comparison between proportions of categories in terms of reports from professionals related to pediatric/adult setting and regional/university hospital. All analyses were two-tailed and conducted at the 5% significance level.

## Results

### Current transition components

Less than half of the respondents (46%) stated that they had a formal transition program and a few (15%) reported that they had a designated transition coordinator. No difference was found regarding the occurrence of a formal transition program in pediatric and adult care (*p* = 0.240). Such programs were more common in university hospitals than regional hospitals (12% vs 2%, *p* = <0.001). Similar results were found for the presence of a transition coordinator (12% vs 3%, *p* = 0.004).

### Destinations of care

**Fig. 1:**
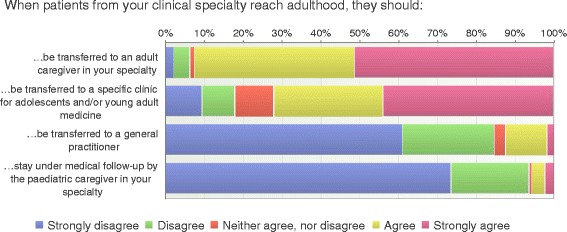
Destinations of care (*n* = 198)

Almost all respondents (94%) were convinced that the young persons should not continue with care within the pediatric care setting nor be transferred to a general practitioner (85%). Almost all (92%) of the respondents expressed a desire for the adolescents to be transferred to adult care within the specialty. A majority (72%) would also consider a specific clinic for adolescents and/or young adults as an appropriate transfer destination (see Fig. 1). Some participants highlighted the need for specific youth clinics to bridge differences in pediatric and adult care:

*It’s good that they know quite a while before that they are going to switch health care providers, but the best would be a youth clinic in which they themselves could decide when they feel ready to move over there, as an intermediate step to adult care.*

### Initiators for transfer

**Fig. 2:**
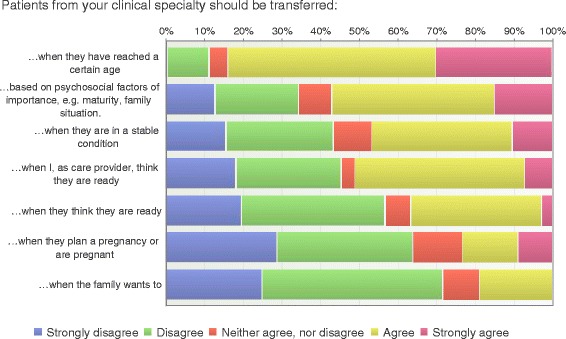
Initiators for transfer (*n* = 197)

**Fig. 3:**
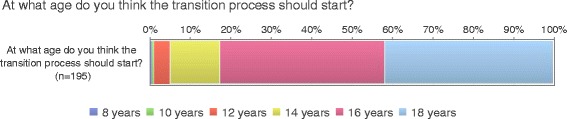
Age for start of transition process

The most significant initiator for transfer was age followed by psychosocial aspects such as maturity and family situations (Fig. 2). Sixteen years of age or older seems to be the preferred age to start the transition process according to 82% of the respondents (Fig. 3). Fifty-seven percent of the health care professionals did not agree that the young person should be transferred when they thought they were ready. Planning for a pregnancy or being pregnant was not considered as an initiator for transfer (Fig. 2).

### Transfer communications

**Fig. 4:**
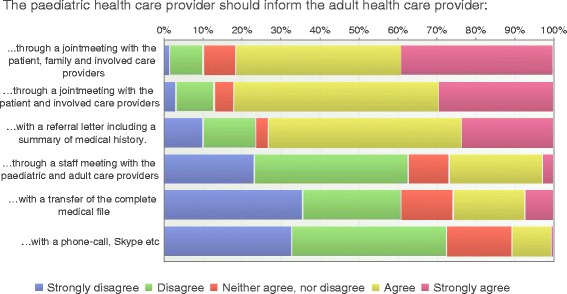
Transfer communication (*n* = 198)

Information from pediatric to adult health care providers during a joint meeting with the patient was preferred by most respondents (82%). Many (82%) thought that the family should be present during the joint meeting as well (Fig. 4), particularly the HCP in pediatric care (73% vs 27%, *p* = 0.03). Only 27% preferred a staff meeting with only pediatric and adult care providers involved showing a significant difference between nurses and physicians (28% vs 72%, *p* = 0.006). Only 11% preferred a telephone call or SKYPE and 26% a transfer of the medical file. One participant in pediatric care described how they concluded the transfer communication with the youth and the adult team as follows:

*We should see the transition as an opportunity to tie loose ends up for the youth. With or without adult colleagues (because they do not feel they have time) I ask about the patient’s experience so far, needs and expectations in the current situation, what they remember of the time with us, how they look at what we’ve done. Talking with them as adults and seeing the individual is an important investment for the future. When the adult team is present, there is so much more to be gained if that knowledge is shared and transferred, and the patient can more easily connect to what’s to come…*

### Participants in transition

**Fig. 5:**
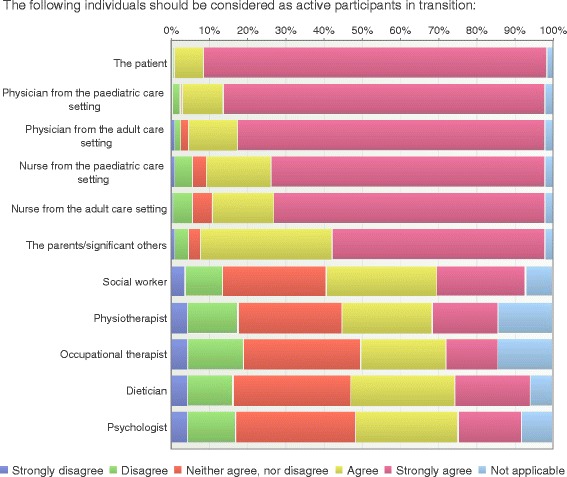
Participants in transition (*n* = 197)

Almost all of the respondents (98%) agreed that the patient should be considered as an active participant in the transition process and 90% agreed that the parents or significant other should be actively involved. The majority of the respondents considered the physicians and nurses to be active participants during the transition process; the physician from the pediatric setting (95%), followed by the physician from the adult setting (93%), the nurse in the pediatric care setting (89%) and the nurse in the adult care setting (87%). Other care professionals were also considered as active participants in the process, for example social worker (52%), dietician (47%), psychologist (43%), physiotherapist (41%) and occupational therapist (36%) (Fig. 5).

### Transition involvement

**Fig. 6:**
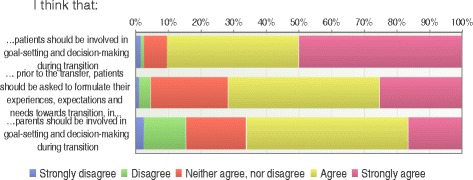
Transition involvement (*n* = 198)

When asked about involvement in the transition process (Fig. 6), 90% considered that patients should be involved in goal-setting and decision-making during transition, while 66% believed that parents should be involved in these issues. HCP in pediatric care preferred to involve parents to a higher degree (89%) compared to HCP in adult care (61%) (*p* < 0.001).

### Patient education

**Fig. 7:**
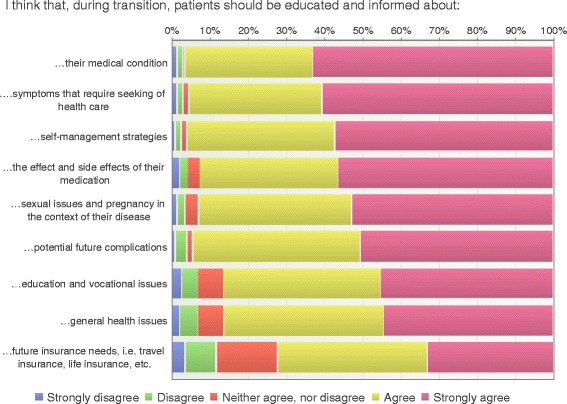
Patient education (*n* = 195)

Most of the respondents considered patient education to be an important task. The information that most respondents considered important concerned the patient’s medical condition (96%), self-management strategies (96%), symptoms that required seeking health care (95%) and potential future complications (94%) (Fig. 7).

*Information and support to develop self-care skills must be given long before the transfer progression. The most important thing is that the patient receives information about their previous treatment, the side effects that can occur, where to turn, but also written information about their past treatment that they can carry with them through life. Preferably with recommendations to health care providers that they may encounter later.*

### Transition barriers

**Fig. 8:**
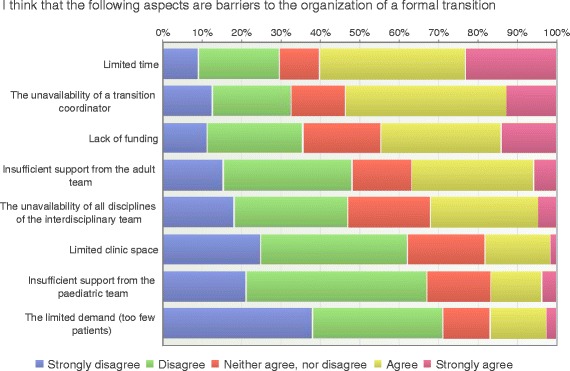
Transition barriers (*n* = 195)

The majority of the respondents (71%) disagreed that limited demand (not enough patients) was a barrier to the organization of a formal transition (Fig. 8). HCP in regional hospitals reported too few patients to be more of a transition barrier than care providers in university hospitals (12% vs 6%, *p* = 0.003). Other aspects of barriers for the organization of a formal transition were limited time (60%), unavailability of a transition coordinator (54%), lack of funding (44%) and limited clinical space (19%) (Fig. 8).

There were significant differences between the reports from care providers in pediatric and adult care regarding insufficient support from adult care (35% vs 8%, *p* = <0.001). This problem appeared to be more prominent in regional hospitals compared to university hospitals (26% vs 18%, *p* = < 0.001). Pediatric care providers professionals also reported the lack of a transition coordinator to be more of a transition barrier than adult care professionals (47% vs 15%, *p* = 0.018). The unavailability of all disciplines in the interdisciplinary team was to a higher degree addressed as a barrier by nurses compared to physicians (58% vs 42%, *p* = 0.02).

### Transition components

**Fig. 9:**
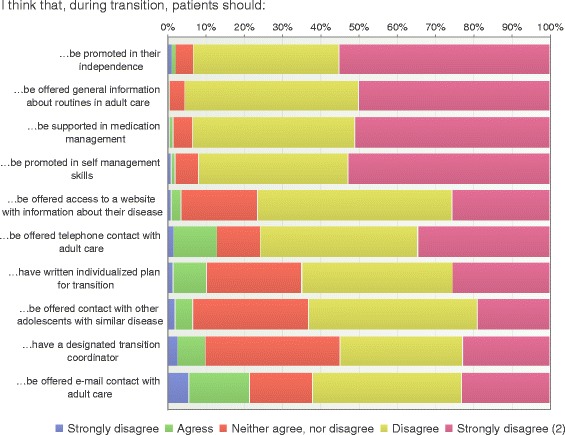
Transition components (*n* = 195)

The majority of the respondents found communication and support during transition to be of importance (Fig. 9). Almost all respondents (95%) believed that adolescents should have contact with peers with a similar disease. Communication included that patients should be offered telephone access (76%); e-mail contacts (62%) and/or access to a specified website with information about their disease (63%). The majority thought that adolescents, during transition, should be promoted in their independence (92%) and self-management skills (93%), while 65% believed that they should be supported in medication management.

A designated transition coordinator was considered an important transition component by the majority of participants (76%) with a difference between HCP in pediatric and adult care (74% vs 26%, *p* = 0.017). Furthermore, 55% agreed that a written individualized transfer plan is an important component and again with difference between pediatric and adult care (76% vs 24%, *p* = 0.004) (Fig. 9).

## Discussion

The main reason for exploring health care professionals’ attitudes to transfer and transition in young people with long-term medical conditions is to gain an overall understanding of issues that may hamper the support of young people during the transition. Using intervention mapping with its ecological approach, the organizational and interpersonal factors are essential in order to improve external validity [3]. Hence, scrutinizing organizational factors and surveying health care professionals attitudes towards transition components might clarify potential pitfalls in the transition process, and increase the likelihood of successful implementation of transition programs.

The current study showed that the absolute majority of HCP in pediatric and adult care wanted patients to be transferred to adult care within their specialty, and they did not consider primary care providers to be active participants in the transition process. Sweden differs from many other countries when it comes to using primary care providers for follow-up of long-term conditions that is congenital or has its debut during childhood. This may not reflect the situation in other countries, which have implications for the generalizability of these findings. This was however, also reported by Hilderson et al. [6], although almost a quarter of centers in their study did not transfer their patients. A somewhat more surprising finding in our study was that many care professionals also acknowledged a designated youth clinic for young persons with chronic diseases as an alternative. Specialized youth clinics for young persons with chronic diseases are still in its infancy in Sweden.

Not surprisingly, age and maturity were reported as the most significant initiator for transfer. The preferred age to start the transition process was 16 years or older. However, this might indicate that the respondents did not distinguish between transition and transfer, where the age for starting the process was more likely related to the actual transfer process, i.e. the change of care provider. This is supported in a meta synthesis by Fegran et al., [9] who found age to be the parameter for transfer, although only half of the included studies explored the issue of age at transfer. Internationally, transfer is generally planned at the age of 18 and centers with a more flexible policy use the age range of 15 – 18 years [10]. However, studies exploring adolescents’ view indicates instead that readiness and maturity are more important than biological age. [11, 12]

A subject for debate when discussing transfer communication with adult health care is the value of joint meetings at time for transfer. The majority of respondents in the current study preferred joint meetings with HCP from both settings, and where both the patient and family are involved. A challenge in making joint meetings routine could be that the pediatric and adult care settings need to be located near each other and preferably in the same hospital, which is not always the case. However, using modern techniques such as teleconference or Skype could be an option, particularly for regional hospitals due to longer distances. This was not supported by the respondents in this study which may reflect an unaccustomed practice of using such media for communication in the health care setting Two thirds of the participants did not prefer staff meetings without the patient and the family present.

It should be noted that we did not ask about the occurrence of joint meetings in the respondents’ organization, but the reports on existing transition programs (46.1%) and having a designated transition coordinator (15.1%) indicate that there is room for improvement in this respect. Both these transitional strategies appeared to be implemented more often in university hospitals than in regional hospitals. This may be related to the fact that staff from regional hospitals reported few patients to be more of a transition barrier than staff in university hospitals. Crowley et al. [13] found successful strategies for transition planning to include joint meetings or transition clinics, supported by studies exploring adolescents’ and parents’ expectations of care [14].

Our findings, showing a positive attitude towards joint meetings and a designated transition coordinator, highlight a willingness to work on strategies that can contribute to better transitional care. Having a transition coordinator who follows up adolescents not attending clinical visits by contacting them has previously proved effective [15, 16]. However, pediatric care providers emphasized parental involvement to a higher degree, reported the absence of a transition coordinator to be more of a barrier but also a more important transition component, compared to HCP in adult care. This resonates a well-known problem, that pediatric HCP ascribe more importance to transitional interventions than adult HCP. This was also supported in our study, in which pediatric HCP considered the support from adult care to be insufficient to a higher extent, than the adult care providers themselves, and especially in regional hospitals. Even if other studies indicate an increased awareness among adult HCP, the identified difference of opinion about the importance of certain transitional strategies in the present study seems to be a crucial barrier for the process [17]. Further, we found that pediatric and adult care providers agreed to a high degree on what aspects of patient education should be included during the transition process. This consensus might facilitate the development of common transitional interventions around educational issues.

### Strengths and limitations

The main strength of this study was its attempt to capture a broad perspective on health care professionals’ attitudes towards transfer and transition in adolescents with various chronic conditions. Although individual needs must be acknowledged, regardless of the type of condition, the description and comparison of transitional issues between different diseases can provide new insights for clinicians across specialties and in both pediatric and adult care settings. A limitation is that, despite our efforts to explicitly distinguish between transition and transfer by defining them within the questionnaire, it became evident that participants were confused by these concepts. It is plausible that this may have affected the clarity of the findings to some point. Another possible limitation is that the questionnaire was tested for content validity but not for other measurement properties such as reliability.

One challenge in this study was the problem of getting access to a national population with representation of HCP in both university hospitals and regional hospitals. We faced many obstacles in the search for nurses and physicians with experience of transfer and transition of adolescents with chronic conditions. Different managers for nurses and physicians, poorly updated e-mail lists, lack of transferred information about the study and its objectives are some examples of such obstacles. These aggravating circumstances are one explanation for the low response rate, but still constitute a limitation of the study. Another explanation might be that HCP who are not particularly involved in either transition or transfer were included in the study due to inadequate selection from the hospital managers. On the other hand, the response rate is still higher or comparable to studies distributing surveys via e-mail. Cunningham et al., [18] among others did not reach a higher response rate from specialized physicians despite providing small financial incentives. In line with these authors we believe that health care professionals may perceive the increasing frequency of surveys as burdensome. However, although the findings may lack generalizability to some extent due to potential responder bias, they do provide some direction for further implementation of transitional care of adolescents with a variety of chronic conditions. To avoid that a transition program remains in researchers’ shelf and in order to facilitate implementation of such programs, the HCPs perspective in planning and developing a transition program is fundamental [3].

## Conclusions

Joint meetings, prior to or at the actual transfer, along with a designated transition coordinator, were reported as important components for transition in this study. The absence of a transition coordinator was also considered to be a transition barrier but less used in current clinical practice. A high degree of consensus appeared regarding patient education and the importance of involving young persons in goal-setting. Despite some differences between the attitudes of HCPs in pediatric and adult care, these findings highlight a willingness to work on transition strategies and provide direction for improvement. Acknowledging important components as well as potential barriers when developing future transition programs can contribute to better transitional care.

## Abbreviations

CHD: Congenital heart disease
CVI: Content validity index
HCP: Health care professionals
IBD: Inflammatory bowel disease
